# Shorter Granulocyte Telomeres Among Children and Adolescents With Perinatally Acquired Human Immunodeficiency Virus Infection and Chronic Lung Disease in Zimbabwe

**DOI:** 10.1093/cid/ciaa1134

**Published:** 2020-08-08

**Authors:** Abhinav Ajaykumar, Glenn C Wong, Louis-Marie Yindom, Grace McHugh, Ethel Dauya, Edith Majonga, Hilda Mujuru, Rashida A Ferrand, Sarah L Rowland-Jones, Hélène C F Côté

**Affiliations:** 1 Department of Pathology and Laboratory Medicine, University of British Columbia, Vancouver, British Columbia, Canada; 2 Centre for Blood Research, University of British Columbia, Vancouver, British Columbia, Canada; 3 Nuffield Department of Medicine, University of Oxford, Oxford, United Kingdom; 4 Biomedical Research and Training Institute, Harare, Zimbabwe; 5 Faculty of Infectious and Tropical Diseases, London School of Hygiene and Tropical Medicine, London, United Kingdom; 6 University of Zimbabwe, Harare, Zimbabwe

**Keywords:** granulocyte telomere length, chronic lung disease, lung function, perinatal HIV infection, combination antiretroviral therapy

## Abstract

**Background:**

Chronic lung disease (CLD) has been reported among African children with perinatally acquired human immunodeficiency virus (HIV) infection (C-PHIV), despite combination antiretroviral therapy (cART). In adults, shorter telomere length (TL) has been reported in association with both CLD and HIV. As little is known in children, our objective was to compare TL in HIV-positive (cART-naive or -treated) and HIV-negative children with and without CLD.

**Methods:**

Participants included Zimbabwean C-PHIV, aged 6–16, who were either newly diagnosed and cART-naive, or on cART for >6 months, and HIV-negative controls of similar age and sex. Packed blood cell (granulocyte) TLs from 621 children were compared cross-sectionally between groups. For a subset of newly diagnosed C-PHIV, changes in TL following cART initiation were evaluated.

**Results:**

C-PHIV had shorter granulocyte TL compared with uninfected peers, regardless of cART. Among 255 C-PHIV without CLD, TL was shorter in cART-naive participants. In multivariable analyses adjusted for age, sex, CLD, and HIV/cART status, shorter TL was independently associated with older age, being HIV positive, and having reduced forced vital capacity (FVC). Last, cART initiation increased TL.

**Conclusions:**

In this cohort, C-PHIV and those with reduced FVC have shorter granulocyte TL, possibly the result of increased immune activation and cellular turnover due to longstanding HIV infection with delayed cART initiation.

## Teaser

Children with perinatally acquired HIV infection (C-PHIV) and children presenting with reduced forced vital capacity (FVC) exhibit shorter granulocyte telomeres. Treatment-inexperienced C-PHIV, especially those with reduced FVC, have the shortest telomeres, and initiation of antiretroviral therapy improved their telomere length.

Despite the success of combination antiretroviral therapy (cART) to prevent perinatal human immunodeficiency virus (HIV) transmission, the most recent estimates from UNAIDS (Joint United Nations Program on HIV/AIDS) suggest that 1.7 million children under the age of 15 years are living with HIV-1 infection, 90% in sub-Saharan Africa (SSA) [1]. In 2018, an estimated 160 000 children acquired HIV-1 [1]. While in the pre-cART era, a majority of children with perinatally acquired HIV-1 infection (C-PHIV) died in early childhood, which access to cART has dramatically improved, it has also been observed that approximately one-third of C-PHIV survive to late childhood and adolescence in the absence of cART [2]. Thus, pediatric HIV programs are now seeing substantial and increasing numbers of older children and adolescents with HIV-1 infection. These young people often experience significant comorbidities [3, 4], affecting multiple systems, which may not improve, especially if cART is initiated later in childhood [5].

The most common comorbidity described in C-PHIV in SSA is a form of chronic lung disease (CLD) [6, 7], typically presenting with a dry cough, reduced exercise tolerance, tachypnea, hypoxia (at rest or following exercise), and progressive impairment of lung function, leading ultimately to pulmonary hypertension and respiratory failure. The first accounts of this condition in C-PHIV came from Zimbabwe [3, 6], but similar observations were subsequently reported in Malawi [7], Kenya [8], and South Africa [9]. In the Zimbabwean ZENITH cohort of 385 children aged 6–15 years newly diagnosed with PHIV (and hence cART-naive), 54% of C-PHIV presented with a chronic cough and 28% had abnormal lung function [4]. Although CLD is more common in untreated older C-PHIV, there remains a substantial burden of disease even among cART-treated C-PHIV, as shown in the INHALE cohort in Zimbabwe, where 25% of C-PHIV on cART had CLD [10].

Chronic immune activation leads to the accumulation of exhausted and senescent cells, which characteristically feature shortened telomere length (TL). Telomeres consist of nucleoprotein complexes that protect the ends of chromosomes. In most somatic cells, TL shortens with each cellular division until a critical point, beyond which cells enter a stage of replicative senescence. In adult populations without HIV, shorter leukocyte TL has been associated with reduced lung function [11, 12], airflow limitation in nonsmokers [13], chronic obstructive pulmonary disease (COPD) [14], and asthma [15]. In the context of HIV, the infection itself is associated with shorter leukocyte TL in adults without and with respiratory diseases [16–19], and in the latter, shorter TL was also measured in small airway epithelial cells [20]. In C-PHIV, 2 studies have reported shorter TL in HIV-infected compared with uninfected children [21, 22], particularly in those not treated with cART [21]. A third study detected no difference but noted shorter leukocyte TL among C-PHIV with a detectable viral load, and TL attrition rate appeared higher among C-PHIV who received cART for less than 15% of their lifetime [23].

In pediatric populations in whom tobacco smoking is a less likely confounder, little is known regarding CLD and TL (both HIV and non-HIV). A large study of participants without HIV suggested a moderate association between shorter leukocyte TL and reduced lung function in adults, but not in children [24]. Knowing that uncontrolled HIV is associated with shorter leukocyte TL and that HIV causes chronic inflammation, which could negatively impact lung health, we hypothesized that C-PHIV with CLD would show shorter TL than non-CLD or non-HIV peers.

## METHODS

### Study Population

**Figure 1.**
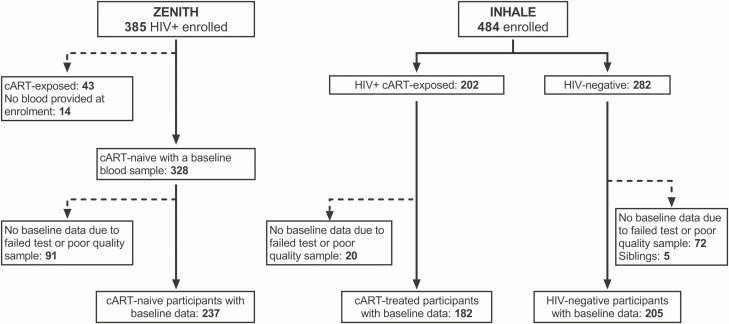
Flow chart of study participants. Telomere length assay did not pass quality control for 1 cART-exposed and 2 HIV-uninfected participants. The numbers of participants with interpretable spirometry test data (lung function test) at enrollment were 133 (HIV-positive cART-naive), 182 (HIV-positive cART-exposed), and 199 (HIV-negative). Abbreviations: cART, combination antiretroviral therapy; HIV, human immunodeficiency virus.

Our study participants included C-PHIV aged 6–16 years enrolled between 2013 and 2016 in 2 previously described cohorts—ZENITH and INHALE [4, 10]. Figure 1 and Supplementary Table 1 provide more information on the study design, study sample, and the 2 cohort studies, including inclusion/exclusion criteria. Briefly, children and adolescents were recruited following HIV diagnosis from 7 primary healthcare clinics (PHCs) in Harare, and were either cART naive (ZENITH), or on cART for more than 6 months (with a median duration of 4.7 years and approximately 80% viral suppression [INHALE]). Children without HIV of similar age and sex were also recruited as controls from the 7 PHCs in Harare [10]. Written informed consent was obtained from the parents/guardians of all study participants. This study was approved by the Medical Research Council of Zimbabwe, the Harare Hospital Ethics Committee, the Biomedical Research and Training Institute Institutional Review Board, and the London School of Hygiene and Tropical Medicine Ethics Committee. Secondary analysis of INHALE and ZENITH participants with available lung function data and blood DNA specimens was undertaken. Demographic and clinical data were obtained from the cohort databases. An HIV plasma viral load (pVL) of more than 50 copies/mL was considered detectable. Cytomegalovirus (CMV) pVL was determined by quantitative polymerase chain reaction (qPCR; Altona Real Star) and more than 1 copy/mL was considered detectable.

### Chronic Lung Disease Classification and Telomere Length Measurement

Lung function spirometry measurements of the highest forced expiratory volume in 1 second (FEV_1_) and forced vital capacity (FVC) were determined for each participant. Measures were categorized as reduced if below the 10th percentile according to the Global Lung Initiative 2012 reference ranges, accounting for height, sex, age, and ethnicity. Chronic lung disease was as described previously [10]—namely, (1) obstruction, defined by reduced FEV_1_:FVC ratio, or (2) reduced FVC, defined as reduced FVC with a normal FEV_1_:FVC ratio.

As peripheral blood mononuclear cells (PBMCs) were not available for most participants, packed blood cells consisting mostly of granulocytes were obtained during blood Ficoll Paque separation (detailed in the Supplementary Data). Telomere lengths in this fraction were measured by monochrome multiplex qPCR as previously described [25]. Relative TL was expressed as a ratio between telomeric DNA (T) and single-copy nuclear gene (S) copies, yielding the T/S ratio. Specimens that did not meet quality-control criteria of a less than 15% difference between replicates over 2 assay attempts were excluded from analyses [25].

### Statistical Analyses

Relative granulocyte TL data were log_10_-transformed for all analyses. Correlations and comparisons of demographic and clinical characteristics as well TLs for the 3 groups were conducted using Spearman’s correlations and Mann-Whitney *U*, Kruskal-Wallis, chi-square, and Fisher’s exact tests. Dunn’s test was used to adjust for multiple pairwise comparisons as appropriate. Factors important univariately (*P* < .10) were considered for inclusion in multivariable analyses of covariance. In sensitivity analyses, extreme TL values that fell outside the 1.5× interquartile range (IQR) for each group and considered biologically implausible were excluded. For a subset of cART-naive C-PHIV who also had a longitudinal specimens available post–cART initiation, intraindividual change in TL was compared using the paired Student’s *t* test.

## RESULTS

### Participant Characteristics

A total of 237 newly diagnosed cART-naive C-PHIV, 182 cART-treated C-PHIV, and 205 HIV-uninfected children were included in this study. Demographic and clinical characteristics of all children are presented in Table 1. There were no differences in age and sex between children in each group. Consistent with a previous report in these cohorts [26], a greater proportion of cART-naive C-PHIV had detectable CMV compared with cART-treated C-PHIV and HIV-uninfected children (*P* < .001) (Table 1). Twenty-one percent (38/182) of cART-treated C-PHIV had an HIV pVL of more than 400 copies/mL at study enrollment, and among the cART-naive C-PHIV, despite no treatment, 19% (45/237) remained undetectable.

**Table 1.** Demographic and Clinical Characteristics of Study Participants at the Baseline Visit

Characteristics	HIV-Positive cART-Naive (n = 237)	HIV-Positive cART-Treated (n = 182)	HIV-Uninfected (n = 205)	*P*
Male sex	115 (49)	94 (52)	98 (48)	.73
Age, y	11 (6–15)	11 (6–16)	11 (6–16)	.68
BMI, kg/m^2^	15.76 (11.49–24.96)	16.05 (10.73–24.96)	16.86 (8.97–31.37)	<.001
Detectable CMV	53 (40) (n = 133)	39 (21)	17 (8)	<.001
CMV viral load, copies/mL	1122 (42–38 190)	1120 (175–106 026)	1220 (402–2703)	.98
Plasma HIV viral load		n = 180		<.001
>400 copies/mL	192 (81)	38 (21)	…	
50–400 copies/mL	0 (0)	18 (10)	…	
<50 copies/mL (undetectable)	45 (19)	124 (69)	…	
HIV viral load, copies/mL	38 074 (3517–939 350)	2254 (55–878 301)	…	<.001
CD4 count, cells/μL	420 (2–2045) (n = 236)	710 (22–1844)	…	<.001
CLD	(n = 133)		(n = 199)	.004
Reduced FVC	22 (17)	21 (12)	11 (6)	
Obstruction	11 (8)	6 (3)	4 (2)	
No reduced FVC or obstruction (CLD-negative)	100 (75)	155 (85)	184 (92)	
Number of household smokers				>.99
0	184 (78)	131 (72)	177 (86)	
1	44 (19)	34 (19)	27 (13)	
2 or 3	6 (3)	6 (3)	1 (1)	
Unknown	0 (0)	11 (6)	0 (0)	
Duration on cART, years	---	4.4 (0.5–12.4)	…	
Percentage of lifetime on cART	0 (0)	44 (4–90)	…	
Type of cART				
NNRTI-based	…	140 (77)	…	
PI-based	…	36 (20)	…	
Other^a^	…	6 (3)	…	
Relative TL		(n = 181)	(n = 203)	.005
Raw	9.66 [8.09–11.69]	9.66 [8.56–11.46]	10.30 [8.80–13.21]	
Log_10_ transformed	0.98 [0.91–1.07]	0.98 [0.93–1.06]	1.01 [0.94–1.12]	

Data are presented as n (%), median (range), or median [interquartile range]. Comparisons were done using Mann-Whitney *U*, Kruskal-Wallis, chi-square, and Fisher’s exact tests as appropriate.

Abbreviations: BMI, body mass index; cART, combination antiretroviral therapy; CLD, chronic lung disease; CMV, cytomegalovirus; FVC, forced vital capacity; HIV, human immunodeficiency virus; NNRTI, nonnucleoside reverse transcriptase inhibitor; PI, protease inhibitor; TL, telomere length.

^a^The list of “Other” cART regimens is detailed in Supplementary Table 2.

Among cART-treated C-PHIV the median (range) duration of cART was 4.4 (0.5–12.4) years, and the percentage of lifetime on cART was 44% (4–90%). The majority (77%) received nonnucleoside reverse transcriptase inhibitor (NNRTI)–based cART (Table 1, Supplementary Table 2) and there were no differences between the average percentage of lifetime treated with NNRTI-based cART (46%) versus protease inhibitor (PI)–based cART (41%).

Lung function data were available for a total of 514 children, of whom 54 (11%) presented with reduced FVC and 21 (4%) with an obstructive spirometry (hereafter referred to as an “obstruction”). The prevalence of both reduced FVC and obstruction was significantly higher in the cART-naive C-PHIV group compared with the HIV-uninfected group; however, there were no differences in CLD status between cART-treated C-PHIV and HIV-uninfected children (Table 1).

### Telomere Length

**Figure 2.**
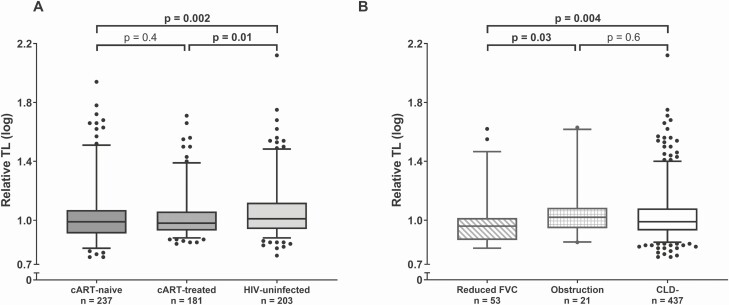
Univariate comparisons of log-transformed relative TL. *A*, cART-naive C-PHIV versus cART-treated C-PHIV versus HIV-uninfected (Mann-Whitney *U* tests). *B*, Children with reduced FVC versus obstruction versus normal lung function (Mann-Whitney *U* tests). For all panels, whiskers of the box plots represent the 5th–95th percentiles. Abbreviations: cART, combination antiretroviral therapy; CLD, chronic lung disease; C-PHIV, children with perinatally acquired human immunodeficiency virus; FVC, forced vital capacity; HIV, human immunodeficiency virus; TL, telomere length.

Granulocyte TL data that passed quality control were obtained for 237 cART-naive C-PHIV, 181 cART-treated C-PHIV, and 203 HIV-uninfected children. In univariate analyses, compared with HIV-uninfected children (median [IQR] log_10_ TL: 1.01 [0.94–1.12]), both cART-naive (0.98 [0.91–1.07]) and cART-treated (0.98 [0.93–1.06]) C-PHIV had shorter TLs (Figure 2*A*), and among the latter, children on NNRTI-based regimens had significantly shorter TLs compared with HIV-uninfected children (Supplementary Figure 1). Sex, age, and number of household smokers showed no univariate association with TL (Supplementary Table 3). Although TL tended to be shorter in children with detectable CMV compared with those with undetectable CMV (*P* = .06) (Supplementary Figure 2), there was no relationship with CMV pVL (Supplementary Table 3).

**Figure 3.**
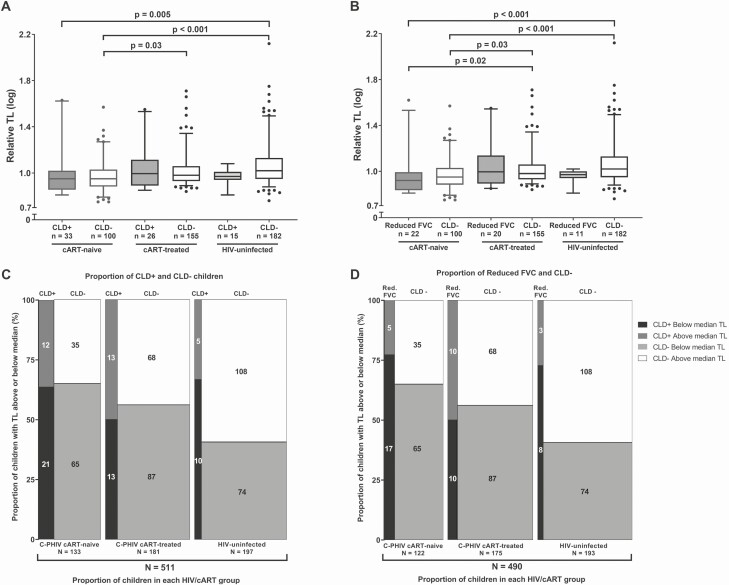
*A* and *B*, Relative TL comparisons between HIV/cART status groups separated by (*A*) CLD+  versus CLD– and (*B*) reduced FVC versus CLD–. For both panels, Dunn’s adjusted *P* values (adjusted for multiple comparisons, Kruskal-Wallis *P* < .001) are shown. Whiskers of the box plots represent the 5th–95th percentiles. *C* and *D*, Mosaic plots showing the relationship between HIV/cART status, above versus below the median TL of the whole cohort (TL = 9.83) and CLD+ versus CLD– status (*C*) or reduced FVC versus CLD– status (*D*). The width of each box shows the proportion of participants for each group relative to the total number of participants for whom both TL and lung disease data were available (n = 511). The height of each box shows the proportion of participants within each group who fall above or below the median TL of the entire study sample. The number within each box represents the actual number of participants for each of these subgroups. Abbreviations: cART, combination antiretroviral therapy; C-PHIV, children with perinatally acquired human immunodeficiency virus; CLD, chronic lung disease; FVC, forced vital capacity; HIV, human immunodeficiency virus; Red., reduced; TL, telomere length.

Overall, children with reduced FVC had significantly shorter TL (median [IQR] log_10_ TL: 0.96 [0.87–1.01]) compared with both children with an obstruction (1.02 [0.95–1.08], *P* = .03) and children without CLD (0.99 [0.93–10.8], *P* = .004) (Figure 2*B*). Based on this observation, in addition to our a priori definition of CLD, we also performed analyses restricted to CLD presenting as reduced FVC (Figure 3*A* and 3*B*). Combination antiretroviral therapy–naive C-PHIV with CLD (median [IQR] log_10_ TL: 0.95 [0.86–1.02]) and without CLD (0.95 [0.89–1.03]) had significantly shorter TL than HIV-uninfected children without CLD who had the longest TL (1.02 [0.95–1.13]) (Figure 3*A*). Among children without CLD, cART-naive C-PHIV had shorter TL (0.95 [0.89–1.03]) than both cART-treated (0.98 [0.94–1.06], *P* = .03) and HIV-uninfected children (1.02 [0.95–1.13], *P* < .001), but there were no differences between cART-treated C-PHIV and the HIV-uninfected group (Figure 3*A*). Similar results were obtained when the analysis was restricted to children who presented with reduced FVC (Figure 3*B*). Our mosaic plot (Figure 3*C*) illustrates that the prevalence of CLD was approximately 25% (33/133) for cART-naive C-PHIV, almost 2 times higher than the CLD prevalence within cART-treated C-PHIV (14%, 26/181), and more than 3 times that of HIV-uninfected children (8%, 15/197). Further, only 35% (47/133) of cART-naive C-PHIV TLs were above the median TL of the overall study sample (median [IQR] log_10_ TL: 0.99 [0.93–1.08]) compared with 45% (81/181) of cART-treated and 57% (113/197) of HIV-uninfected children. Likewise, the prevalence of reduced FVC, as well as the proportion of children with TLs above the study median TL, was very similar to the overall model for CLD (Figure 3*D*).

**Figure 4.**
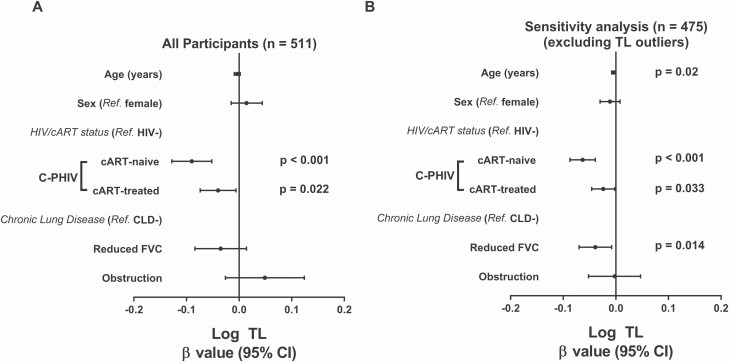
Multivariable regression analyses of the association between possible predictors and log-transformed relative TL. *A*, All participants (n = 511, *R*^2 ^ =  ^ ^0.05). *B*, Sensitivity analysis excluding participants with implausibly high outlying TL values (n = 475, *R*^2 ^ =  ^ ^0.08). Other variables that were considered but were not included in the final model include CMV viral load (detectable vs undetectable) and number of household smokers (any vs none). Abbreviations: cART, combination antiretroviral therapy; CI, confidence interval; C-PHIV, children with perinatally acquired human immunodeficiency virus; CLD, chronic lung disease; CMV, cytomegalovirus; FVC, forced vital capacity; HIV, human immunodeficiency virus; Ref., reference; TL, telomere length.

In a multivariable model adjusted for age, HIV/cART status, and CLD status, only being C-PHIV was independently associated with shorter granulocyte TL (Figure 4*A*). In a sensitivity analysis, 21 cART-naive, 12 cART-treated, and 19 HIV-uninfected participants with implausibly high outlying TL values were excluded. In this model (Figure 4*B*), shorter TL was independently associated with older age, being HIV positive, and having reduced FVC. In secondary models (Supplementary Figure 3*A* and 3*B*), we explored the effect of cART type and observed a modest association between NNRTI-based cART and shorter TL; the effect size was similar for PI-based cART, but the 95% confidence interval was wider due to the smaller number of participants treated with PI. This effect was not related to age or the percentage of life on cART.

**Figure 5.**
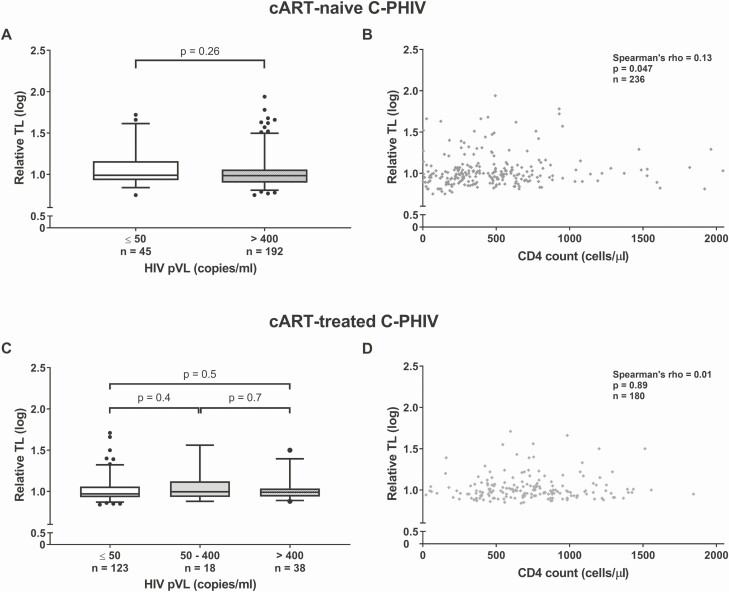
Comparisons showing that viral load and CD4 cell count are not univariately associated with relative TL among cART-naive C-PHIV (*A* and *B*), and cART-treated C-PHIV (*C* and *D*). Univariate comparisons were done using Mann-Whitney *U* tests and whiskers of the box plots represent the 5th–95th percentiles. Similar results were obtained after adjusting for age and percentage of lifetime on cART (for cART-treated C-PHIV) through multivariable models (data not shown). Abbreviations: cART, combination antiretroviral therapy; C-PHIV, children with perinatally acquired human immunodeficiency virus; HIV, human immunodeficiency virus; pVL, plasma viral load; TL, telomere length.

**Figure 6.**
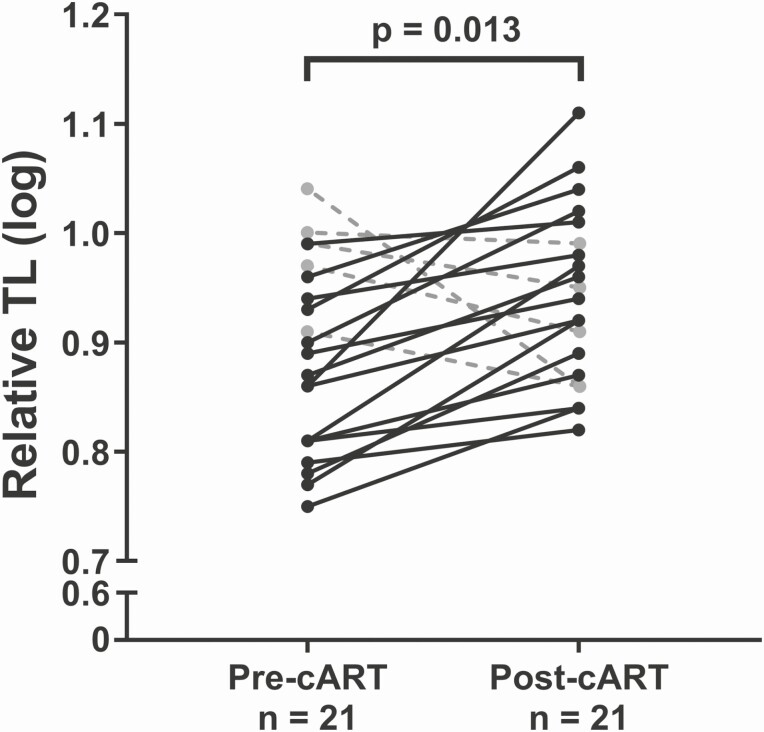
TL increases following cART-initiation in cART-naive C-PHIV. Solid black lines indicate participants for whom TL increased and dashed gray lines indicate participants for whom TL decreased post–cART initiation. Comparisons were done using paired Student’s *t* test. Abbreviations: cART, combination antiretroviral therapy; C-PHIV, children with perinatally acquired human immunodeficiency virus; TL, telomere length.

Last, we did not observe any associations between TL and HIV pVL (Figure 5*A* and 5*C*) or CD4 count (Figure 5*B* and 5*D*) obtained from plasma collected at the same time point as DNA collection among C-PHIV, irrespective of cART status. However, for a subset of cART-naive C-PHIV (n = 21) who had a second specimen available post–cART initiation, a longitudinal increase in TL (*P* = .013) was observed (Figure 6).

## DISCUSSION

As reported previously [10], a high prevalence of CLD is observed among Zimbabwean C-PHIV, despite cART. Given the known relationships between HIV, TL, and lung disease in adults, we tested the hypothesis that CLD among C-PHIV would be associated with shorter TL, compared with non-CLD or non-HIV peers. We report shorter granulocyte TL among C-PHIV, regardless of cART status, as well as among children presenting with CLD defined by reduced FVC. Telomere length was shortest in cART-naive C-PHIV with CLD and appeared to improve following cART initiation in a subset of children.

Our findings are in contrast with the only other study of leukocyte TL and lung function in children, which reported no association between TL and any spirometry index of lung function among 11-year-old children [24]. However, that study took place in a high-income setting (Australia), measured TL in all blood cells, and included only HIV-uninfected children, making a comparison between the 2 studies difficult.

The underlying pathogenesis of CLD in older children and adolescents with PHIV is unknown and this study was not designed to examine potential mechanisms. However, in this sample, our observations of shorter granulocyte TL only among children presenting with reduced FVC may indicate a relationship with restrictive rather than obstructive lung disorder [27]. Telomere length shortening in this case could be a result of increased immune activation and cellular turnover in response to HIV and/or CLD disease, something that has been reported in studies of adults with HIV [18, 19]. In contrast to the pre–antiretroviral therapy era, where the majority of CLD was a result of lymphocytic interstitial pneumonitis, high-resolution computed tomography studies of adolescents with PHIV and CLD in Zimbabwe and South Africa have revealed radiological features of mosaic attenuation and air trapping consistent with obliterative bronchiolitis (OB), with or without bronchiectasis [28–30]. While OB is most commonly seen following lung or hematopoietic stem cell transplantation in developed countries, the pathogenic mechanisms underlying this condition in African children with PHIV remain unclear, with persistent immune activation and chronic inflammation the most likely driving factors. In fact, outside the transplant population, OB has principally been described following severe lower respiratory tract infections in young children, often with adenovirus, and appears to be more common in the southern hemisphere [31].

Cytomegalovirus coinfection represents an important cofactor implicated in the development of HIV-associated comorbidities [32] and may play a more important role in African children, most of whom become infected with CMV in early life [33]. For children diagnosed late with PHIV, it is likely that primary CMV infection occurred in infancy at a time of uncontrolled HIV replication—hence, could substantially contribute to comorbidities in this group. In keeping with this hypothesis, we recently described unexpectedly high levels of CMV viremia in older children and adolescents with PHIV in Zimbabwe, even in those who were treated with cART [26]. Furthermore, detection of CMV viremia at levels above 1000 copies/mL was associated with reduced lung function in cART-naive children. In the current study, where the vast majority of children would be expected to be CMV seropositive, we observed no relationship between CMV pVL and granulocyte TL. It is likely that the frequency of CMV reactivations may modulate TL in these children, but longitudinal CMV viremia data were not available.

Within C-PHIV, TL was longer and the prevalence of CLD was lower among cART-treated children compared with cART-naive children, suggesting that treatment may be beneficial for both lung health and cellular aging. Compared with 2 other studies of TL in pediatric PHIV [21, 23] that included younger children, our study participants had slightly lesser cART exposure in terms of percentage of life on cART. It is possible that the observations of shorter TL among both cART-naive and cART-treated C-PHIV, although modest, may become more pronounced later in life when the cumulative burden of HIV/cART and other environmental factors such as exposures to household smoke and other particulate pollutants have taken effect. A recent study from South Africa reported persistently lower lung function over 2 years among cART-treated adolescents living with HIV compared with HIV-negative controls [34]. Here, longitudinal CLD data were not available but we observed an improvement in TL following cART initiation in cART-naive children, although it remained lower than in HIV-uninfected controls. As such, while cART may not necessarily improve lung function in older C-PHIV, longitudinal studies extending into adult life would help ascertain TL dynamics in the context of CLD severity/disease progression after an extended period of viremic control.

Our study has several strengths and some limitations. The ZENITH and INHALE cohorts included a well-characterized group of older children with PHIV, both treated and untreated, as well as HIV-negative control children of similar age and sex from the same population. This enabled us to delineate the effects of uncontrolled/untreated HIV on CLD severity and cellular aging. Furthermore, in adult studies reporting TL in lung disease, tobacco smoking, a factor well established to be associated with shorter TL [35], is often prevalent and an important confounder. Our investigation among children, in whom smoking is uncommon, allows a more robust analysis of the relationship between TL and lung disease. A major limitation of our study is the fraction of blood cells from which TL was quantified, which mostly consisted of neutrophils, an uncommon cell subset for telomere studies. Although we cannot ascertain that the TLs measured here reflect overall PBMC or leukocyte TL for these children, several studies suggest a strong correlation between TL measured in neutrophils and either PBMCs, T lymphocytes, or leukocytes [36–38]. While it is possible that our finding is limited to granulocytes, immune activation is heightened systemically during chronic HIV and increased stimulation of granulocytes by microbial translocation occurs even in the absence of viremia [39]. Furthermore, neutrophilic inflammation has been implicated in HIV and CLDs such as asthma, COPD, cystic fibrosis, and bronchiectasis [40], and therefore telomere dynamics in these cells may be relevant, potentially reflecting shorter telomeres in progenitor cells. Last, we did not have access to lung tissue from participants with CLD and could not measure TL in lung cells. Future studies investigating TL in lung tissue and leukocytes could help corroborate our findings in granulocyte TL and ascertain the systemic nature of CLD.

In conclusion, treatment-inexperienced, older African C-PHIV exhibited the shortest granulocyte TL and a higher prevalence of CLD in this cohort. Among children with CLD, only those presenting with reduced FVC had shorter TL, suggestive of increased cellular aging in relation to a restrictive lung disorder in these children. Last, cART initiation in treatment-naive children appears to improve TL. Taken together, our results suggest that cART treatment is protective against lung disease and cell aging in C-PHIV.

## Supplementary Data

Supplementary materials are available at *Clinical Infectious Diseases* online. Consisting of data provided by the authors to benefit the reader, the posted materials are not copyedited and are the sole responsibility of the authors, so questions or comments should be addressed to the corresponding author.

ciaa1134_suppl_Supplementary_Data

ciaa1134_suppl_Supplementary_Figure_S1

ciaa1134_suppl_Supplementary_Figure_S2

ciaa1134_suppl_Supplementary_Figure_S3

ciaa1134_suppl_Supplementary_Legends

## Notes

***Acknowledgments.*** The authors thank all the study participants and the research staff at the Biomedical Research and Training Institute in Harare.

***Disclaimer.*** The funding sources had no role in the writing and submission of the work described above.

***Financial support.*** This work was supported (in part) by a Canadian Institutes of Health Research team grant (in Cellular aging and HIV comorbidities in women and children) (grant number TCO-125269; to H. C. F. C.). A. A. was partly supported by a UBC Centre for Blood Research Internal Collaborative Training Award. G. C. W. was funded by an A Star graduate scholarship from the Singapore Agency for Science, Technology, and Research. L.-M. Y. was funded by the International AIDS Society Collaborative Initiative for Paediatric Human Immunodeficiency Virus Education and Research Programme (grant number 2016/345-YIN) and the Royal Society of Tropical Medicine and Hygiene. R. A. F. was funded by the Wellcome Trust (grant number 095878/Z/11/Z). S. L. R.-J. received funding from the International Research Centre for Medical Sciences, University of Kumamoto, Japan.

***Potential conflicts of interest.*** The authors: No reported conflicts of interest. All authors have submitted the ICMJE Form for Disclosure of Potential Conflicts of Interest.
