# Supplementary material for: Shorter Granulocyte Telomeres Among Children and Adolescents With Perinatally Acquired Human Immunodeficiency Virus Infection and Chronic Lung Disease in Zimbabwe
Source: Clin Infect Dis. 2020 Aug 8;73(7):e2043–51. doi: 10.1093/cid/ciaa1134 (PMC8492138; doi:10.1093/cid/ciaa1134)
Supplement: ciaa1134_suppl_Supplementary_Data [file ciaa1134_suppl_supplementary_data.docx]

**SUPPLEMENTARY DATA**

**Whole blood collection, PBMC preparation, and packed cell layer separation**

Fresh whole blood (~12 mL) was collected in Sarstedt EDTA tubes and centrifuged at 1,500 rpm for 5 mins to separate the plasma layer. Plasma was transferred to fresh Sarstedt tubes (1.5 mL each) and stored at -80^o^C until required. The remaining blood fraction was diluted 1:1 with RPMI-1640 media and mixed gently. Approximately 9-10 mL of diluted blood was layered on ~4 mL of Ficoll solution (ficoll: blood ratio of 1:2), and specimens were then centrifuged at 400g for 25 mins with brakes OFF. Following centrifugation, the PBMC fraction was carefully separated (taking as little Ficoll solution as possible) and transferred to 50 mL Falcon tubes for subsequent live cell PBMC storage. The remaining packed cell layer above the erythrocyte layer was carefully separated for subsequent DNA extraction.

**SUPPLEMENTARY TABLES**

**Table S1.** Cohort descriptions, enrollment period, and inclusion/exclusion criteria

| **Cohort** | **Cohort description** | **Inclusion criteria** | **Exclusion criteria** |
| --- | --- | --- | --- |
| **ZENITH** | Study participants were enrolled in 7 public sector primary healthcare clinics (PHCs) in 7 high population density suburbs in southwest Harare, Zimbabwe between January 2013 and December 2014 | Children aged 6-15 years, newly diagnosed with HIV infection, residing in and planning to receive HIV care in one of the 7 study communities | Children residing outside Harare, as follow-up may have been difficult due to distance to travel. |
| **INHALE** | Study was carried out between September 2014 and December 2016 at the Harare Children’s Hospital HIV clinic in Harare, Zimbabwe – a public sector clinic that provides HIV care for more than 4000 children and serves the same catchment population as those served by the ZENITH cohort PHCs | **Children with HIV infection:** Children aged 6-16 years, taking antiretroviral therapy for at least 6 months, not acutely unwell and not taking tuberculosis (TB) treatment  **HIV-uninfected children:** Children aged 6-16 years, who had tested HIV-negative, who were not acutely unwell, and who were not receiving treatment for respiratory infection or TB. HIV-uninfected children were recruited from the same 7 PHCs as HIV-positive children. Mothers of HIV-uninfected children were not tested for HIV. | None |

**Table S2.** List of “other” cART regimens

| Regimen | # of children |
| --- | --- |
| AZT+FTC | 1 |
| TDF+FTC | 1 |
| AZT+TDF+FTC | 2 |
| AZT+FTC+NVP+ATZ | 1 |
| DDI+ATZ+EFV | 1 |

AZT – Zidovudine, FTC – Emtricitabine, TDF – Tenofovir disoproxil fumarate, NVP – Nevirapine, ATZ – Atazanavir, DDI – Didanosine, EFV – Efavirenz

**Table S3.** Univariate analyses of the association between possible predictors and log-transformed relative TL for all study participants

| Explanatory variables | r_s_^a^ | p value |
| --- | --- | --- |
| Male sex (ref. Female) | --- | 0.29 |
| Age (per year) | -0.07 | 0.08 |
| Detectable CMV (ref. undetectable CMV) | --- | **0.06** |
| CMV VL (copies/ml) | 0.001 | 0.99 |
| CLD status: Reduced FVC *vs.* Obstruction *vs.* CLD- | --- | **0.012^b^** |
| Reduced FVC *vs.* CLD- | --- | **0.004** |
| Obstruction *vs.* CLD- | --- | 0.63 |
| Reduced FVC *vs.* Obstruction | --- | **0.03** |
| # of household smokers (Any *vs.* None) | --- | 0.61 |

^a^Spearman’s rho, ^b^Kruskal-Wallis p value. All other comparisons used the Mann-Whitney U test

CMV – cytomegalovirus, CLD – chronic lung disease
