# Supplementary figures and images for: Shorter Granulocyte Telomeres Among Children and Adolescents With Perinatally Acquired Human Immunodeficiency Virus Infection and Chronic Lung Disease in Zimbabwe
Source: Clin Infect Dis. 2020 Aug 8;73(7):e2043–51. doi: 10.1093/cid/ciaa1134 (PMC8492138; doi:10.1093/cid/ciaa1134)

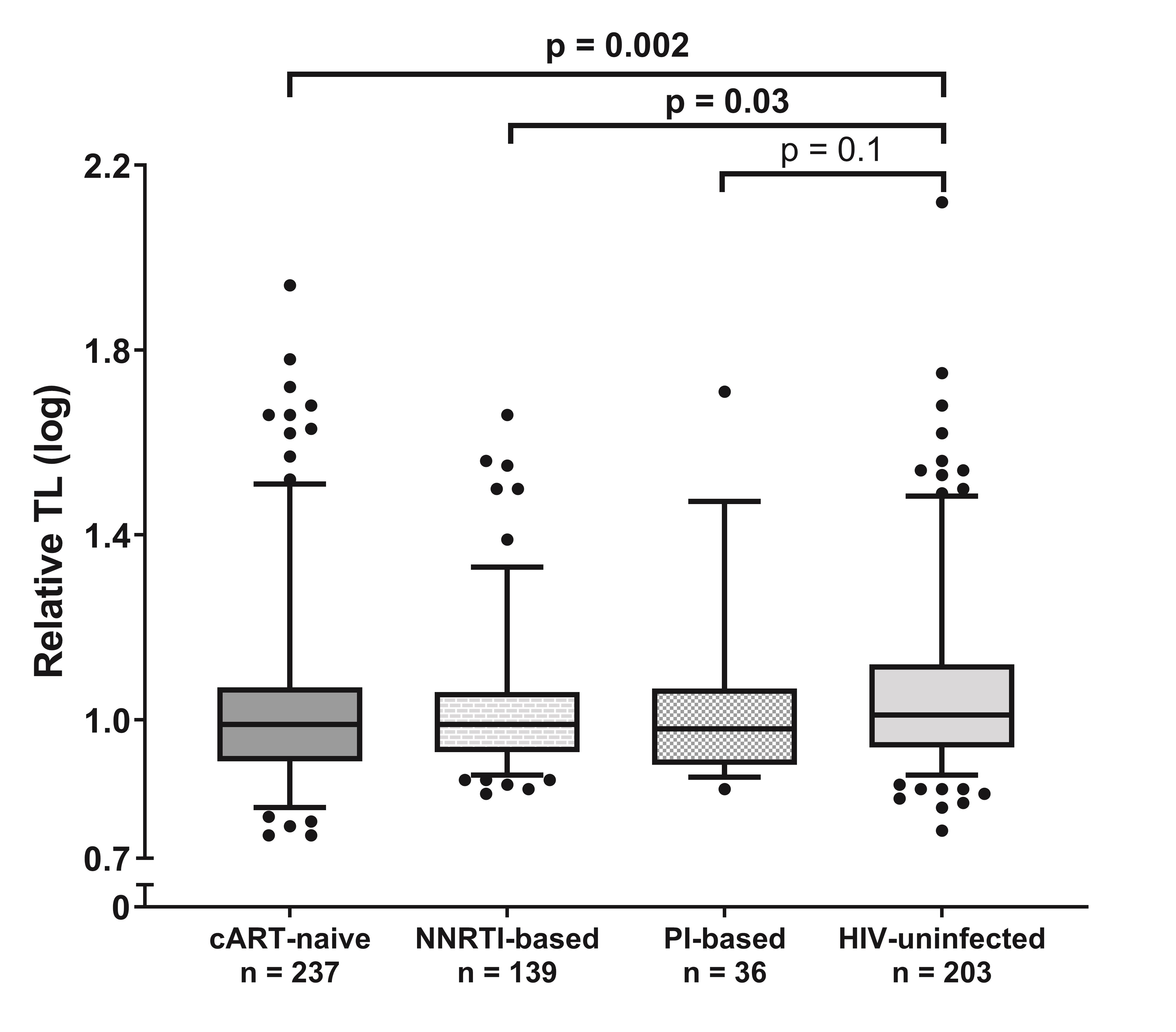

Supplement: ciaa1134_suppl_Supplementary_Figure_S1 [file ciaa1134_suppl_supplementary_figure_s1.jpeg]

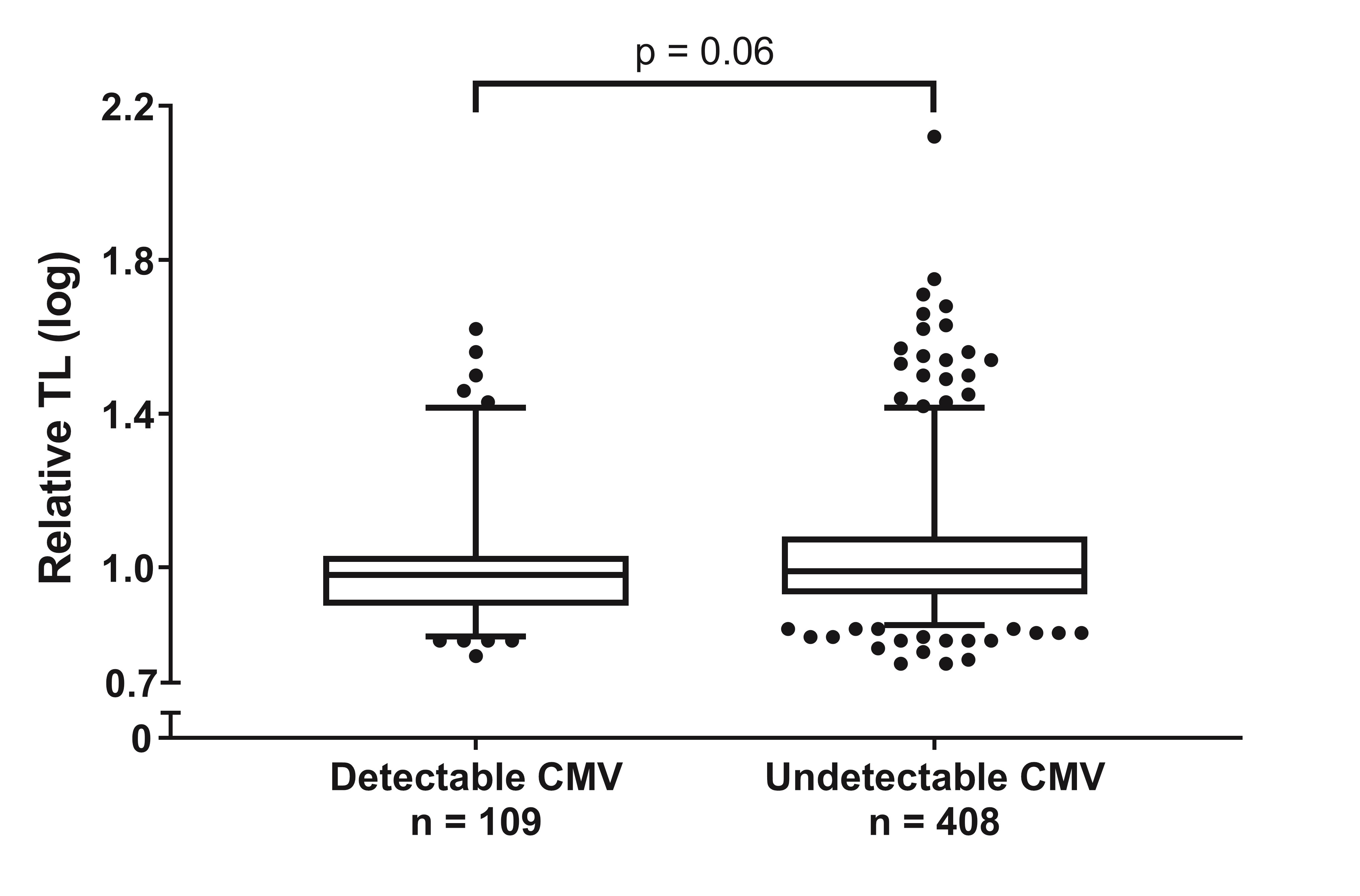

Supplement: ciaa1134_suppl_Supplementary_Figure_S2 [file ciaa1134_suppl_supplementary_figure_s2.jpeg]

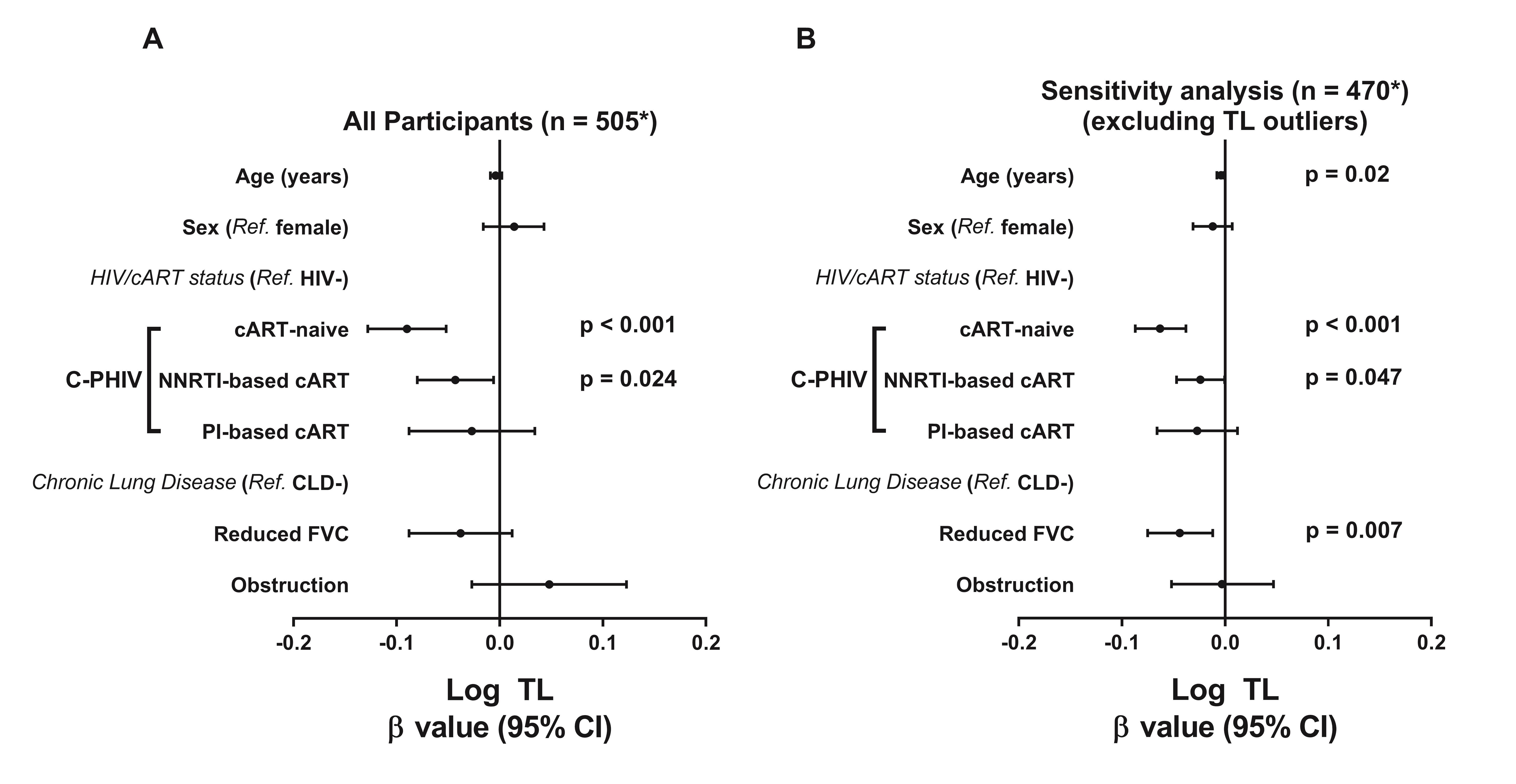

Supplement: ciaa1134_suppl_Supplementary_Figure_S3 [file ciaa1134_suppl_supplementary_figure_s3.jpeg]
