# Supplementary material for: Shorter Granulocyte Telomeres Among Children and Adolescents With Perinatally Acquired Human Immunodeficiency Virus Infection and Chronic Lung Disease in Zimbabwe
Source: Clin Infect Dis. 2020 Aug 8;73(7):e2043–51. doi: 10.1093/cid/ciaa1134 (PMC8492138; doi:10.1093/cid/ciaa1134)
Supplement: ciaa1134_suppl_Supplementary_Legends [file ciaa1134_suppl_supplementary_legends.docx]

**FIGURE LEGENDS**

**Supplementary Figure S1. Univariate comparisons of log-transformed relative TL** between cART-naïve C-PHIV, NNRTI-based cART-treated C-PHIV, PI-based cART-treated C-PHIV, and HIV-uninfected children (Mann-Whitney U tests). Whiskers of the box plots represent the 5-95 percentiles.

**Supplementary Figure S2. TL are not different univariately between children with detectable and undetectable CMV.** Comparisons were done using Mann-Whitney U tests and whiskers of the box plots represent the 5-95 percentiles.

**Supplementary Figure S3. Multivariable regression analyses of the association between possible predictors and log-transformed relative TL: A –**all participants, with cART-treated C-PHIV separated by type of cART regimen (n=505, R^2^ = 0.06). **D –**sensitivity analysis, with cART-treated C-PHIV separated by type of cART regimen (n=470, R^2^ = 0.09). *6 children who received non-standard cART (detailed in Supplementary Table S1) were excluded from these analyses.
